# Saturn and its Rings: Four Centuries of Imperfect Amodal Completion

**DOI:** 10.1177/2041669518822084

**Published:** 2019-01-21

**Authors:** Sergio Roncato

**Affiliations:** Dipartimento di Psicologia Generale, Università di Padova, Italy

**Keywords:** cognition, perception, perceptual organization, segmentation, spatial cognition

## Abstract

The planet Saturn is a familiar image for us, but it presents perceptual peculiarities that impeded the discovery of its structure and which can still be misleading today. Saturn appears to be surrounded by rings which hide it to a certain extent and then continue behind the outline of the planet. What we perceive is the result of a double amodal completion in which the planetary globe and the rings exchange the roles of occluding and occluded surface. Saturn was hidden to 17th-century astronomers for half a century because their rudimentary telescopes did not reveal the pictorial clues that are fundamental for discovering such a complex perceptual organization as that formed by a globe surrounded by rings. Moreover, the existence of a celestial body of this nature was inconceivable in light of the knowledge of those times. The improvement of telescopes has substantially enriched our knowledge of Saturn, but historic documents highlight the importance of perceptual organization factors. Astronomical observations were a rich source of information, but only Huygens was capable of integrating them and hypothesizing the true structure of Saturn. His drawings are the result of a particular ability to integrate observations and also the ability to use pictorial information. It is likely that these diagrams were an important instrument for the solution. The image of Saturn, however clear, will not find universal consensus. The planet has “residual (perceptual) capacities” of hiding itself which can even deceive modern instruments. Here, we will try to understand why.

*The length of time between the first Galileo’s observations of Saturn’s rings and Huygens correct explanation was due in part to the poor resolution of the early telescopes. However, a great difficulty was recognition of the possibility and plausibility of astrophysical disk system. Contrast this to the situation today, when almost any flattened object observed in the heavens is suspected of being a disk. (* *Lissauer, 1989* *, p. 1)*

## Introduction

The organization of the perceptual world is the outcome of processes of integration, grouping, and meaning attribution. We distinguish two broad categories to indicate whether cognition is involved or not. Early processing gives rise to instantaneous effects, autonomous perceptual units, or structures because these are not susceptible to cognitive influence. Top-down or conceptual-driven processes act as interpolating and structuring factors obeying to learned rules or laws, reasoning, recognition, or retrieval from memory.

Amodal completion can be the result of both types of integration processes. An outline interrupted or erased for a certain length becomes something continuous if the gap is filled by a contour or surface; the figural integration is the outcome of basic processing. Kanizsa (1979) illustrates with astonishing examples the strength of these phenomena and their “resistance” to the influence of cognition. The latter, in turn, may generate strong completion effects that manifest themselves, for example, during reading. The printed words may be visible only in part, such as in “ai#port,” but we do not hesitate in reading “airport”; it is our linguistic knowledge that allows us to complement the series of letters with the missing “r.”

Following Kanizsa, we will refer to these classes of interpolation phenomena—line completion and word integration—as primary and secondary “ways of going beyond the information given.”

In ordinary experience, early processing and conceptually driven processes interact in various ways. They may concur or collaborate. Primary and secondary interpolations tend to act in accordance with natural situations as when we move or drive a car. When they conflict, the object becomes instable or vanishes as occurs in camouflage. Episodes such as “inattentional blindness” (Mack & Rock, 1998) highlight an abnormal prevalence of the secondary processes that manifest a structuring power comparable to the primary ones.

If the two processing modes are hard to coordinate under routine conditions, one wonders what happens in exceptional situations. To initiate explorations means, for example, to venture into an unknown environment in which the stimuli have abnormal intensity and amount, where cognition cannot compensate for poverty of stimulation and the stimuli are too vague to retrieve a suitable conceptual structure. The perceptual organization and understanding face the combined action of two obstacles.

This is an exceptional circumstance but is not such a rare event in scientific exploration. One of these events, or better an era of explorations, occurred in the first half of the 17th century and has as its protagonists Saturn, astronomers, and the just invented telescope. The astronomers, first among all Galileo, who pointed their telescopes toward the planet, saw a luminous shape surrounded by ill-defined contours, an appearance that changed in time with a variability that did not allow the recognition of an object or celestial body. In these five decades of exploration, we have the firsthand accounts of the astronomers and the remarkable work of historians. It is the story of a wholly elusive object: a source of imprecise and poor information, unrecognizable, and unclassifiable.

The pictorial and written reports of the observers are therefore an important piece of evidence of how, in “unknown” circumstances, they go beyond the information given. Gregory (1970) take them as an example of “compositions of stored object-hypotheses” (p. 120), that is beliefs in what the objects ought to be. Pirenne (1970) draws on the same reports to demonstrate how preconceived ideas influence our perception and scene understanding.

In the first phase, when telescopes were rudimentary, primary processes played an important role. We can recognize their action in astronomers’ sketches where, for example, contour completion is something added to the rough images. This interpolation effect may combine with secondary processes, preconcepts, or wrong inferences.

The improvement of telescopes has provided more detailed images, but the solution to Saturn’s enigma did not result from the more accumulation of knowledge. Huygens, the Dutch discoverer, could have used a more powerful instrument but what allowed him to follow a more productive procedure was his way of data processing.

Historians highlight his ability in using Cartesian logic. Here, we will try to show how figural processing and pictorial data interpolation has played a supporting role, if not a decisive one.

They may be partly related even to the “resistance” by the contemporaries to accept the Huygens’ model. The image of Saturn is the result of a double amodal completion in which the planetary globe and the rings exchange the roles of occluding and occluded surface. Nevertheless, the rings are not a good candidate for the role of occluding figure; some of them (e.g., the innermost one) are translucent, and they may be indistinguishable from their cast shadows on the planet. They give rise to misleading optical effects that are not avoidable even by means of the most advanced instruments of astronomical observation.

**Figure 1.**
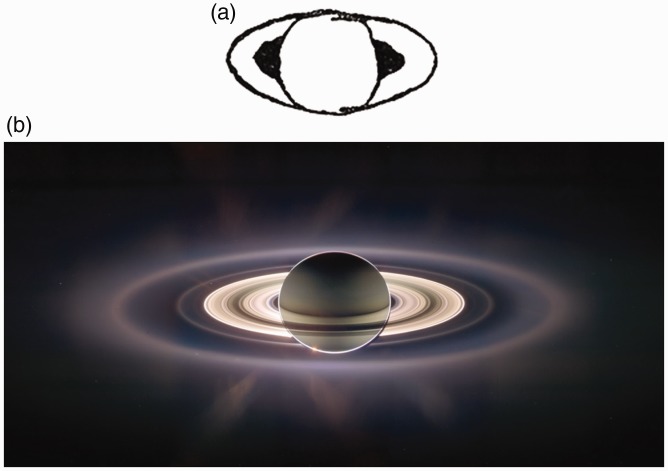
(a) Galileo’s sketch of Saturn in 1616 and (b) Saturn’s eclipse released by NASA. NASA description*:* *This marvelous panoramic view was created by combining a total of 165 images taken by the Cassini wide-angle camera over nearly 3 hours on September 15, 2006. The full mosaic consists of three rows of nine wide-angle camera footprints; only a portion of the full mosaic is shown here. Color in the view was created by digitally compositing ultraviolet, infrared, and clear filter images and was then adjusted to resemble natural color. The mosaic images were acquired as the spacecraft drifted in the darkness of Saturn’s shadow for about 12 hours, allowing a multitude of unique observations of the microscopic particles that compose Saturn’s faint rings (https://www.nasa.gov/mission_pages/cassini/multimedia/pia08329.html)*.

In Figure 1(a), we have reproduced a Galileo’s sketch of what he saw when focusing his telescope toward Saturn. In Figure 1(b), a photo released by NASA shows a Saturn eclipse as seen from the Cassini spacecraft some years ago. These two images impress for an analogy: The globe appears as superimposed on the rings. The planet “resists” the occlusion and appears in a mistaken position despite the huge progress in the astronomic observations clarifying how perceptual factors are involved in this misperception is the main aim of this work.

With the aid of the excellent work of science historians (Andriesse, 2004; Beima Martini, 1842; Boschiero, 2007; Buonanno, 2014; Del Prete, 2008; Fletcher, 2004; Grego & Mannion, 2010; Howard, 2004; Price, 2000; Shapley, 1949; van Helden, 1968, 1970, 1974a, 1974b, 1984, 1994, 2004, 2006), we have retraced the main steps of Saturn’s first explorations.

## Galileo: The First Images of Saturn

Saturn was visible to the naked eye long before the invention of the telescope. In 1610, Galileo discovered some surprising images, thanks to his opportunity to use this instrument. Saturn appeared to him as a central sphere with large moons on either side, but 2 years later in 1612, when the rings turned edgewise to the earth, the lateral bulges disappeared, an image that left the great scientist bewildered. “Has Saturn swallowed his children?” he wrote, invoking the ancient myth of Cronus (Saturn) eating his newborn children. The bewilderment and acknowledgment of the inability to understand the phenomenon persisted when the “strange appendages” reappeared in 1616 (see Figure 1(a)). Below is the passage in which he informs Federico Cesi, an aristocrat from Umbria, who founded the first academy of sciences in Italy (Accademia dei Lincei).

*I don’t want to keep from telling Your Excellency of a new and strange phenomenon, which I observed several days ago about the star of Saturn, whose two companions are no longer two small, perfectly round globes as they were before, but are at present much larger bodies, and no longer round, as seen in the adjoined figure, 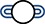, that is, two half eclipses [* *sic* *] with two dark little triangles in the middle of the figures, and contiguous to the middle of Saturn, which is seen, as always, perfectly round. (Galileo, translation by* *van Helden, 1974b* *, p. 109).*

The sketch in the text is drawn by the person who copied the letter. Historians agree that it is the sketch in Figure 1(a), and printed in the Assayer (Il Saggiatore), that was referred to by Galileo.^1^

Is there a complete correspondence between this sketch and what Galileo perceived in September 1616 through the lenses of his telescope? Did Saturn appear as segmented into two subunits as in Figure 1(a) or is this drawing the result of a perceptual organization?

The roughness of the telescope could not allow to see the outline contours of the drawing, they are graphic expedients to reproduce in the white sheet a configuration made up of a circular shape and two half eclipses at its opposite sides. In doing so, how much did Galileo go “beyond the information given”? A question far too risky and naive if addressed to the one who highlighted in his writings the deception of the senses (Piccolino & Wade, 2008).

However, we can determine how far the two images diverge by comparing the image projected by the telescope and the configurations drawn in Figure 1(a).

**Figure 2.**
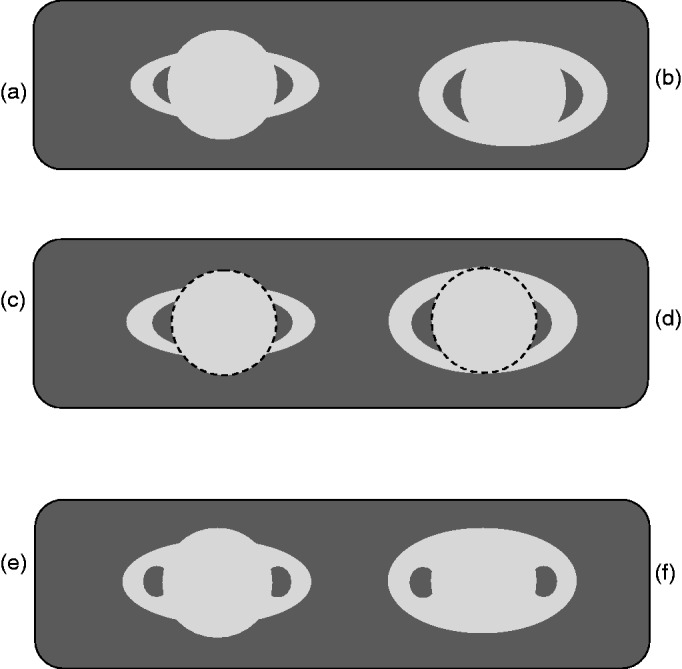
(a and b) Saturn, in two different rings inclinations, reproduced as an isochromatic surface. (c and d) The amodal completion. A dashed circumference symbolizes the stratification of the images into a disk and superimposing to the ellipsoids. (e) and (f) reproduce (a) and (b) with the slices rounded.

In this website (https://brunelleschi.imss.fi.it/telescopiogalileo/etel.asp?c=50021), we can get an idea of what Galileo perceived through the lenses of his telescope: light, fuzzy contoured ovoid figures with some shadow spots. Yet no cues can be singled out that allow the division of these light shapes into components: In order to perceive a disk occupying the central region, some form of figural completion segmentation must occur, for example, the curved upside border is to be seen to fill a gap and prolong following a circumference (see Figure 2).

We can speculate that it is this modal completion that has led Galileo to draw a central circled outline in Figure 1(a). Yet he does not report a corresponding percept of amodal completion that should lead him to describe an ellipsoid behind the circle. Instead, he indicates “half-ellipses.” Some historians see in Figure 1(a) an ellipsoid crown occluded by the planet, which they see as proof that Galileo was the first to observe Saturn’s rings. An opposite view is held by van Helden (1974a):

*Although the improvement of the telescope was certainly one factor contributing to the solution of the problem, it is simply wrong to speak of the “resolution of the so called* *ansae* *or ‘handles’ into one encircling ring by Huygens.” The figures drawn, for example, by Galileo in 1616 and Divini in 1649, show that when the ring was in its most open aspect with respect to the Earth, telescopes were good enough, at a very early stage, to show something which a modern observer could easily interpret as a ring. But neither Galileo nor Divini (nor anyone else) made the “gestalt switch” from “seeing” a globe with two attached “handles” to seeing a globe surrounded by a ring. (p. 155).*

Half a century ago, these words of van Helden opened up interesting prospects for psychology, albeit somewhat late, but we will use this opportunity to test the psychology of perception as a tool to understand the evolution of world knowledge.

What prevented the “gestalt switch”? The planet Saturn is a familiar image for us, but for those who have no idea of this pattern, it is hard to “extrapolate” a correct image from a vague representation. For this to be possible, the globe and the ring must emerge as distinct unities and in a precise depth order. The globe hides the rings with one of its hemispheres and is hidden by the same rings on the opposite hemisphere. This depth order inversion must be “computed” on the basis of perceptual cues, the T-junctions, that emerge only in a sufficiently detailed image. These cues could not be captured by the first telescopes. Therefore, we can distinguish two phases in Saturn’s exploration by astronomers. Observers could see uniform, isoluminant shapes interrupted by vague shadows in the first phase due to the low resolution of telescopes. A second phase followed with the improvement of telescopes which began to provide detailed images of the planet with the T-junctions.

## Figural Completions in the First Telescopic Images (Telescopic Images and Amodal Completion)

As we have already claimed, the images observed through the first rudimentary telescopes, starting with Galileo’s exploration, were hard to process, but some form of interpolation and inferences left some traces in astronomers’ reports. With the aid of two replicas of Saturn’s image, we can illustrate two configurations that originate from different segmentation and completion processes. They are not yet the “gestalt switches” that van Helden expected, but they are still a perceptual organization, something “beyond the telescopic information.”

In Figure 2, a pair of “telescopic” images of Saturn are depicted in the top row. They are not an exact replica of what a 20-power telescope, such as that used by Galileo, allowed to capture, but they serve to illustrate how these light silhouettes against a dark background can organize perceptually. The two images differ in the inclination of the rings, which is close to the maximal tilt in the right-side image, and intermediate in the left one.

The same pair of figures is depicted in the row below (c and d) with a dashed central circumference to indicate the modal completion of the globe. This modal completion of the globe in front and amodal completion of the rings behind it, rather than vice versa, are predicted by Petter’s (1956) rule that in these circumstances the self-split of the light image into a circled surface and a curved stripe behind is predicted.

The layered percept—globe above the ellipsoid—is very likely to be perceived in Figure 2(a) due to the short gap and the collinear L-junctions; such conditions are absent in Figure 2(b) where the modal completion of a central shape is very uncertain. An alternative perceptual organization emerges in the form of an ovoid surface with a pair of dark slices in front of it. This amodal completion stands out as a stable figure-ground organization as soon as the slices are rounded, as the bottom pair of images (Figure 2(e) and (f)) illustrates.

**Figure 3.**
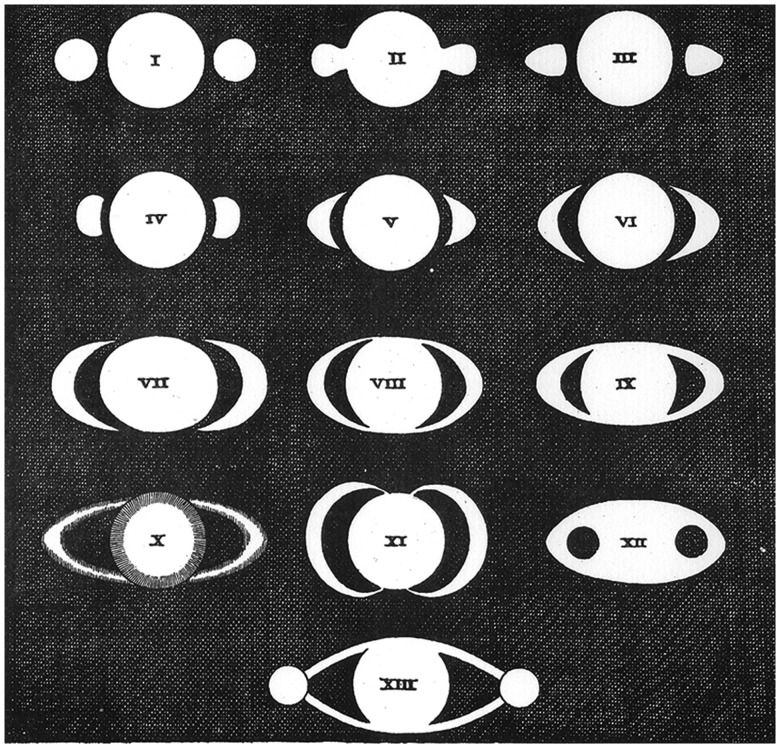
Huygens’ (1659) compendium (p. 32), see https://galileo.ou.edu/exhibits/system-saturn.

The intense astronomic exploration of the first decades of the 17th century confirms that the two perceptual completions illustrated in Figure 2 have “shaped” the figures drawn by astronomers to illustrate what they saw through the lenses. The figural completion in Figure 2(c), hypothesized above for Galileo’s sketch of Saturn, may have influenced the images that contemporary astronomers drew to illustrate the planet. Figure 3 reproduces the compendium created by Huygens (1659) in his *Systema Saturnium* to give a synthesis of the images of Saturn drawn by different observers from 1610 to 1646. Note the presence of the central disk in almost all the sketches, a demonstration that the completion of the central figure persists despite the different appearances of the rings. The drawing with the roman numeral X, reproducing an engraving of Divini, is the most eloquent demonstration of the amodal completion of the rings and the modal completion of the globe.

Let’s look at the drawing with the roman numeral XII. It reproduces one of the numerous figures that the French astronomer Gassendi (1683, cited in Shapley, 1949) drew to illustrate his telescopic observations. One can recognize the amodal completion that occurs in Figures 2(e) and (f) when the slices or shadows are rounded: They emerge as figures against a background filled by the propagation of the light areas.

## A Data-Driven or Conceptual-Driven Completion?

The hypothesis that the images of Saturn are the outcome of modal and amodal completions does not exclude that secondary processes, such as logic inferences or preconcepts, had some involvement. For example, the “satellite model,” that is the presence of a planet and one or more orbiting celestial bodies, may have conditioned observers’ analyses of the telescopic images. Saturn appeared to Galileo in the first observation (1610) as a sphere with two small circular bodies at the opposite sides; a pattern fully congruent with the “satellite model” and likely to have influenced his successive observations. The fragment of the letter we reported before leaves no doubt. Galileo singles out a “globe … which is seen, as always, perfectly round.” The presence of a globe is taken for granted and its perfect roundness as well. The lateral figures are “contiguous” that is something adjacent, not occluded by, the central disk. To conclude, in Figure 1(a), the influence of primary and secondary interpolations is not easily distinguishable from each other. Perhaps the true misleading event is the convergence of the two processes: The central globe is originated by modal completion and at the same time is predictable on the basis of the “satellite model.” Nevertheless, all the other astronomers who followed Galileo in his astronomic exploration collected so many and different images of Saturn that neither the “satellite pattern” nor the alternative models could give a meaning to the different appearances of the planet. As one can see in Figure 3, the “gestalt switch” is far from being attained. Whether additional information or a *gestalt-like* restructuring are needed will be examined in the following sections.

## New Telescopes

In Huygen’s table, the drawings of Saturn do not contain outline contours, shadows, or other perceptual cues needed to guess the planet’s configuration. One can illustrate these cues with the aid of outline sketches.

**Figure 4.**
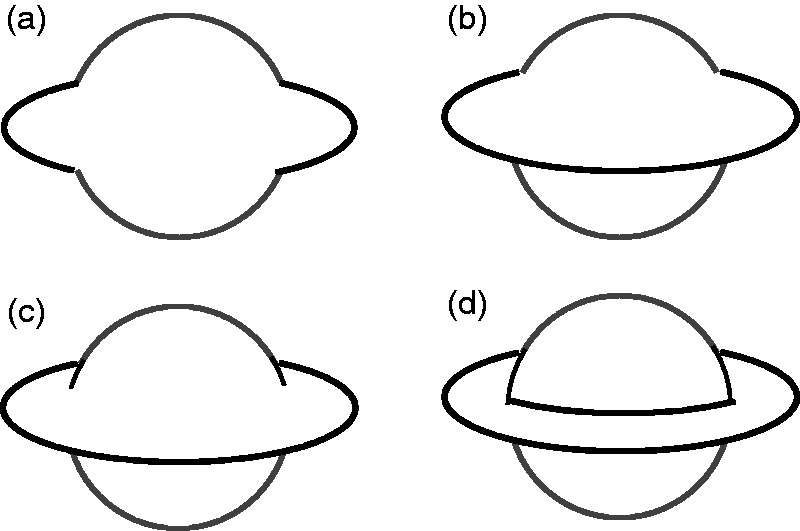
(a) The perimeter of a circle and a superimposed isochromatic ellipse; (b) the same as (a) with two T-junctions; (c) and (d) appearance of further T-junctions.

The progression from Figure 4(a) to (d) shows a gradual appearance of occlusion cues, the T-junctions. Only the complete reproduction of T- and L-cues (Figure 4(d)) allows to perceive the double amodal completion of globe and ring. Two pairs of T-junctions are needed: a pair informing of the globe occlusion and another pair—of opposite depth order—informing of the ring occlusion. Two L-junctions can produce a higher level of realism.

If the telescope image is not detailed enough, this essential information is missing and the topology of the system is a mystery. Galileo’s telescope could magnify objects 20 to 30 times, but this increased toward the mid-17th century with Huygens’ telescopes providing magnifications of 50 and 100 times. Although the appearance of new details did not correspond to an approach to the solution, it added new reasons for conflict between astronomers. Boschiero (2007) gave the title “The War of Telescopes” to his essay on this historical period. Historians provide us with an accurate reconstruction of the progress made in Rome by Divini and Campani as the most important protagonists in the manufacturing of telescopes (Buonanno, 2014; Del Prete, 2008). The dispute also involves Huygens who had published the correct image of the planet in his *Systema Saturnium*. Let us interrupt the history of the conflict and examine his discovery.

## Huygens: Saturn Completion, Conceptual Driven

The first veridical images of Saturn are drawn by Christiaan Huygens, a Dutch mathematician and astronomer, and published in his *Systema Saturnium* (1659). In this essay, a real chronicle of its astronomical exploration, he indicates March 25, 1656, as the day in which he comes to assess the true nature of “Saturn’s appendages”:

*Annulo cingitur tenui, plano nusquam cohaerente, ad eclipticam inclinato. (p. 47)*

*Surrounded by a thin flat ring, nowhere touching, and inclined to the ecliptic.*

Huygens had initiated a systematic observation program the preceding year (1655), claiming that it was grounded on theoretical as well empirical bases.

*Hosautem reputare illum oportet non ex mera inventione atque arbitrio meo hanc me fingere hypothesin, sicut Astronomi sui hepicyclos, nusquam in caelo apparentes; sed oculorum sensu quo nempe reliquarum rerum omnium figuras dignoscimus, hunc quoque annulum satis evidenter me percipere. (p. 48)*

*These men should consider that I do not construct this hypothesis from pure invention and out of my own fancy, as the astronomers do their epicycles, which nowhere appear in the heavens, but that I perceive this ring very plainly with the eyes; with which, obviously, we discern the figures of all other things. (translation by J. H. Walden, see* *http://www-history.mcs.st-andrews.ac.uk/Extras/Huygens_Saturn.html* *)*

It is not clear whether Huygens perceived the Saturn “system,” that is whether his powerful telescope allowed to single out the crucial T-junctions. According to Shapley (1949), the tube’s length allowed to correct the spherical and chromatic aberration. But van Helden (2004) claims that Huygens could not see the rings, “but he did not find it by seeing a ring through his telescope: there was no ring to be seen. No, he found it by seeing a ring in his mind’s eye” (p. 16). Then, the question arises, “How did he arrive to the ring-hypothesis”? (van Helden, 1970, p. 113).

Huygens highlights two crucial observations. The lateral bodies did not change form during a short period of observation in which the planet revolved: Only a circular body “would always present the same aspect to us.” Second, the changes in width in long periods of the appendages are a consequence of the planet revolution and the inclination of the rings with respect to the ecliptic (the whole Huygens’ account is given in Notes section^2^).

According to van Helden (1970, 1974b), three phenomena headed Huygens in the right direction:

- The “anses” (lateral appendages) preserved their length despite narrowness. A single celestial body (not satellites) can generate this image.
- The globe rotation was computed as more rapid than the change of the “anses.” Saturn’s rotation was calculated in an interval between half a day and 16 days, a period in which the “anses” did not show variations. Only a rotating disk can generate this image (van Helden, 1970, 1974b).
- Dark band across the globe (edge-on) that Huygens (erroneously) interpreted as the ring contour.
*Whether this realization came to him as a sudden insight or as the fruit of trial-and-error considerations is not revealed by the author. (* *van Helden, 1974a* *, p. 161)*

The application of the Cartesian method (Miner, Wessen, & Cuzzi, 2007) and the scrupulous annotation of astronomic observations by Huygens support the hypothesis that the solution was the final step of an inferential procedure. However, *Systema Saturnium* leaves open the possibility that some pictorial information allowed Huygens to come to an insightful solution. It is true, as van Helden (1970, 1974a, 1974b) claims, that for several months before March 1656, the rings were edge-on and therefore invisible from the Earth, but the young Dutch astronomer turns out to be a keen observer in poorly informative contexts. In the next paragraph, I shall try to demonstrate how a good use of few perceptual cues contributed to the solution.

## The Huygens’ Drawings and the Visual Solution of Saturn’s Mystery

In the diary of observations of *Systema Saturnium*, we can find significant evidence of how the drawings and the pictorial cues might have influenced and promoted the solution process.

### The First Observations

**Figure 5.**
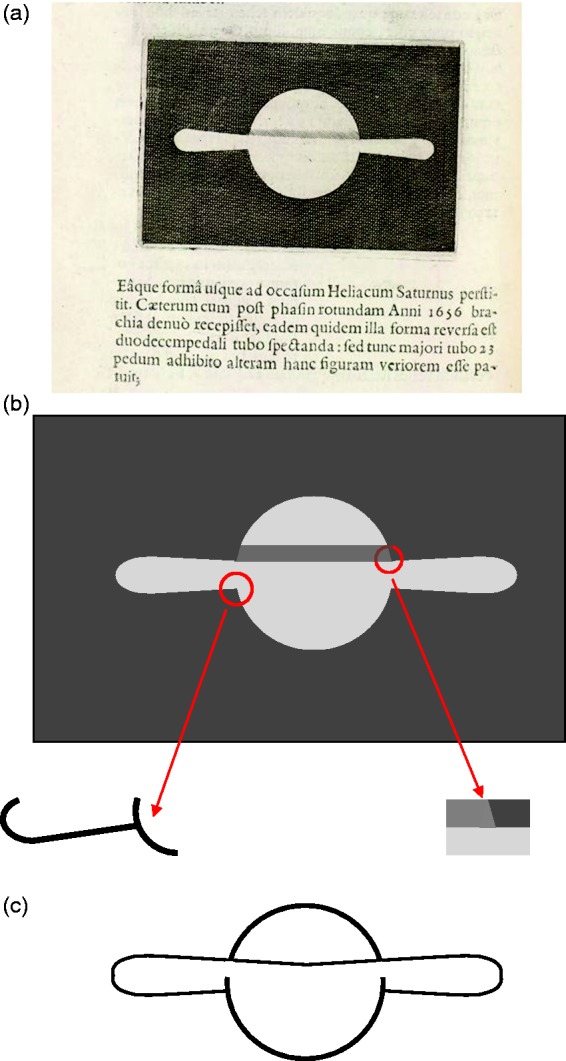
(a) From *Systema Saturnium*. Huygens’ observation of March 1655. Lateral bodies or “brachia” (“arms”) are thicker at their ends. (b) encircled the L-junctions (leftist) and the T-junctions. (c) The hypothesis put forward here is that the L and T-junctions action as depth cues: the upper hemisphere is perceived as occluded, whereas the lower hemisphere is perceived as occluding. The L-junction is assumed to become a “degenerate” T-junction with the arcuate side prolonging to fill the gap in the planet contour.

Huygens indicates March 25, 1655, as the starting date of his astronomic exploration of Saturn. In Figure 5(a), one can see the sketch he drew to reproduce what he perceived when he directed his 12-foot telescope to the planet. He describes the image as a disk with lateral appendages bulging at the extremities and arranged along a straight line. These lateral bodies are named “brachia” (arms). A dark line conjoins the upper contours of these arms.

### The Saturn “Solitary Phase.”

Date of observation: January 16, 1656. Huygens draws an image of Saturn deprived of the lateral appendages an crossed by an equatorial dark band.

These two images are the pictorial information that, combined with the astronomic measures collected by Huygens, allowed him to formulate the ring hypothesis. Nevertheless, the successive progresses in his exploration are of great concern, as they can show the contribution of the pictorial cues and of the phenomenological attributes.

### An Improved Telescope

**Figure 6.**
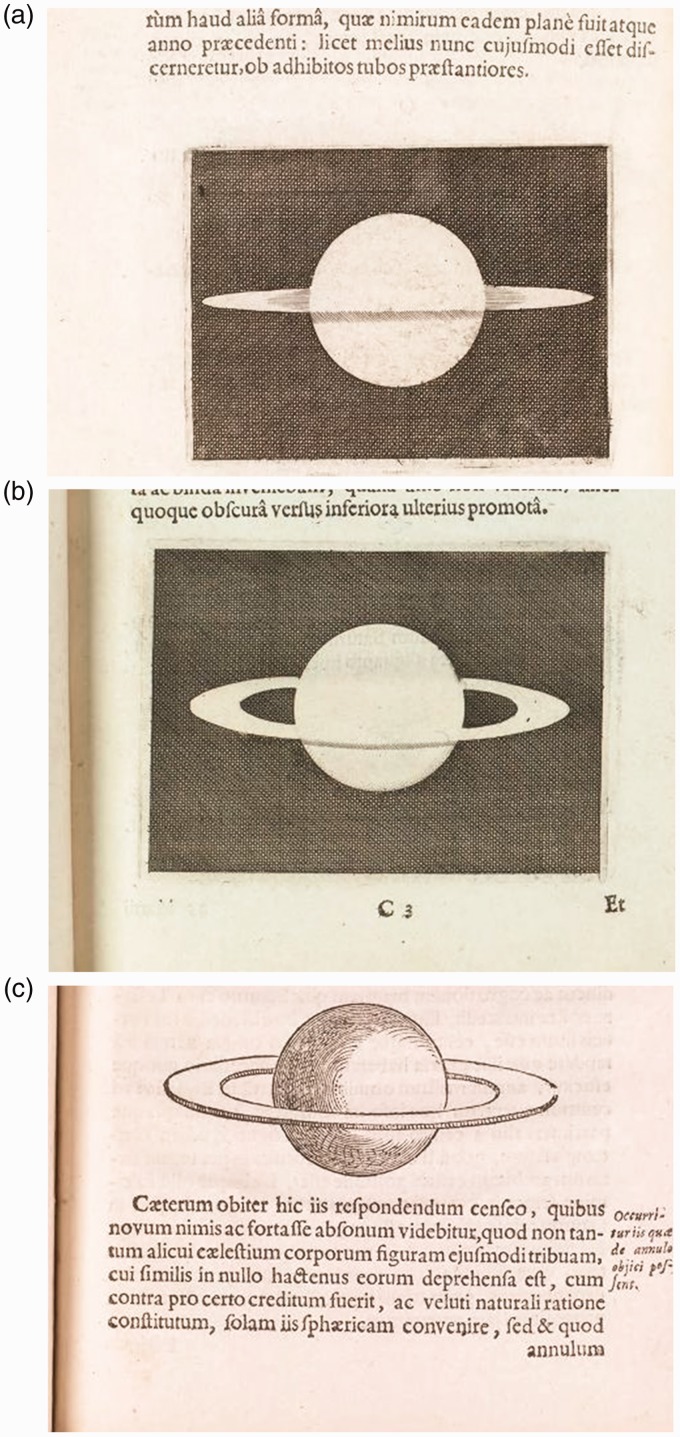
From *Systema Saturnium*. (a) 1656 (October): Saturn seen with a larger telescope (23-feet focal length). The image confirms the hypothesis formulated 6 months before. (b) 1657 (December): The arms (brachia) widen to form a fork or “handles” (ansiae).

The astronomic exploration continued with a 23-foot tube; the first of the new images dated October 13, 1656. It is reproduced in Figure 6(a). The lateral bodies are cusp-like shapes with light shadows in the region adjacent to the planet. These lateral bodies are conjoined by a dark line prolonging their lower contour.

A telescope of equal power focused on Saturn 20 months before, Huygens claims, would have let to see the same image in Figure 6(a) with the shadowed line in the higher hemisphere.

Therefore, the dark equatorial line in Figure 6(a) corresponds to the dark stripe connecting the lateral bodies in Figure 5(a).

### Brachia Saturni in Ansa Mutari (Saturn Arms Change Into Handles; p. 21)

Huygens sketches the image of Saturn seen in 1657 (Figure 6(b)) in which the lateral appendages “bifurcate,” the light shadows of Figure 6(a) now appear as portions of the dark space so that the ring, as a body “nowhere touching” the globe, results more clearly defined.

### The Solution

In Figure 6(c), Huygens draws the image of Saturn as he assumed in his ring hypothesis of March 1655.

Huygens could see Saturn with the lateral bodies (*brachia*) just from the first observations, that is a year before formulating the correct hypothesis . However, Figure 5(a) shows the lateral appendages, the central disk, and the equatorial shadow forming an organized figural pattern with a clear stratification in depth: The horizontal dark strip looks like the shadow cast by a surface superimposed to the equatorial region of the central circular shape. This depth effect (Mamassian, Knill, & Kersten, 1998) vanishes in the lower half. Here, a self-splitting phenomenon seems to emerge: The circular contour tends to fill the gap and conjoin with the arc nearby. In Figure 5(b) and (c), the enlargements illustrate the sources of the stratification effects. A pair of T-junctions in the upper half sum their effects with the shadow-like band to make the central region to appear in relief. This depth order reverses in the lower half where a pair of L-junctions are assumed to act as “degenerate” T-junctions (see Figure 5).

It may be hypothesized that this raw image generated an important suggestion for the Dutch astronomer. We cannot know whether it had a determinant role or, on the contrary, it was a visual representation of a solution obtained by an inferential procedure. Furthermore, we cannot exclude that it is the result of a posteriori reconstruction of the astronomic observations of 1655 to 1656.

We limit ourselves to highlighting that the main features registered in the first observations, that is, the lateral bodies and the equatorial dark band, combine in the Huygens’ drawing to form an image in which the Saturn mystery is solved: The two component bodies and their spatial relations are almost entirely evident.

In Figure 6, one can see the sketches that Huygens drew to represent his observations from October 1656 (6 months after the solution) to 1659, year of publication of *Systema Saturnium*. It is surprising that a realistic and detailed image of the system planet rings (Figure 6(c)) is created 3 years after the formulation of the hypothesis in which all the main features of the surrounding ring were described: thin, flat, nowhere touching. Huygens behaves as if he hesitated to publish the veridical image of Saturn preferring to show the preparatory efforts to come to a solution, that is the “approximations” sketches of Figure 6(a) and (b).

An answer can be found in Huygens’ words illustrating Figure 6(c):

*Thus the true appearance is such as I have indicated in the appended scheme. I believe that I should digress here to meet the objection of those who will find it exceedingly strange and possibly unreasonable that I should assign to one of the celestial bodies a figure the like of which has up to this time not been found in any one of them, … (p. 47; translation by J. H. Walden).*

Figure 6(c) is a “schema”: The pictorial representation of a definition not an image captured through the lenses of a telescope. Such a detailed image will be seen with Campani’s (1664) telescope some years later. But Huygens is aware that it will be greeted with skepticism and disbelief. His hypothesis, nonetheless, has an important support: The images sketched in Figures 5(a) and 6(a and b). Despite the incomplete set of T-junctions, the stratification in depth is evident and the double amodal completion as well.

The solution of Saturn’s mystery is a *visual solution* emerging from the phenomenological organization of the depicted: junctions, shadows, and contours.

Did they promote an insightful thinking or are the conclusive step of an inferential procedure? It is impossible to give a clear answer. We can only assume that such self-explanatory images of the unknown planet have had a remarkable importance in promoting and supporting the intellectual “adventure” of the young Dutch astronomer.

## A Misleading Amodal Completion

**Figure 7.**
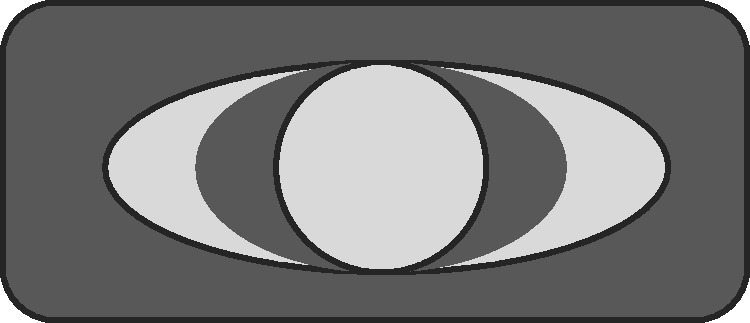
Saturn drawn by Gassendi. From Huyugens: *Systema Saturnium*.

In the same years, Gassendi made the drawing reproduced schematically in Figure 7. No T-junction appears that could suggest veridical amodal completions. The whole configuration that emerges, that is, a disk, a dark ellipse, and a larger one ordered in depth, demonstrates that the amodal completions occurred but with a very different result from the veridical one.

We cannot conclude that Gassendi was misled by a wrong or too rough a sketch as he collected images of Saturn that were very different to Figure 7; nevertheless, the fact remains that his knowledge and logical skills, not inferior to Huygens’, did not lead him to a solution. The same goes for Hevelius, Wren, and Roberval, who had the observative data and logical resources to discover the rings of Saturn but failed to do so.

It is likely that Huygens looked with greater intent at what other observers might have considered an irrelevant pictorial detail or a trick or deception.

Some events are significant in this regard because they highlight the “resistance” to Huygens’ “ocular” evidence. The war of telescopes continued. The Dutch scientist did not limit himself to put forward a hypothesis and successfully tested the predictions about Saturn’s appearance at different times. This, however, was not enough to gain widespread support. Rival theories such those of Hevelius and Wren (Bennett, 1975) demonstrated good predictive power as the dispute that opposed Huygens on one side and Fabri and Divini on the other reached its peak. French Jesuit Fabri, on the basis of the observation of telescope maker Divini, claimed that Saturn had two pairs of satellites: two of them dark and moving in a closer orbit and the other two bright and moving in a farther orbit. In 1660, the Accademia del Cimento was charged to settle the dispute. This provided an important chance to plan an experiment: A model of Saturn was built (according to Huygens’ designs) and observed through telescopes of differing strength from a distance of about 75 m. The instruments provided all the changing images that were gathered by several astronomers during the preceding decades. Fabri admitted his errors and apologized to Huygens.^3^

The resolution of the dispute by the Accademia del Cimento did not generate a general agreement on Huygens’ hypothesis and the criticism continued even when telescope maker. Campani published the results of the observations with a “state-of-the art” telescope confirming Huygens’ hypothesis. Campani’s image is clear and the ring encircling the globe unequivocal, yet it is to be demonstrated whether an outstanding scientist like Hooke could analyze the planet with an inductive procedure.

## Hooke: Saturn as Data-Driven Completion

**Figure 8.**
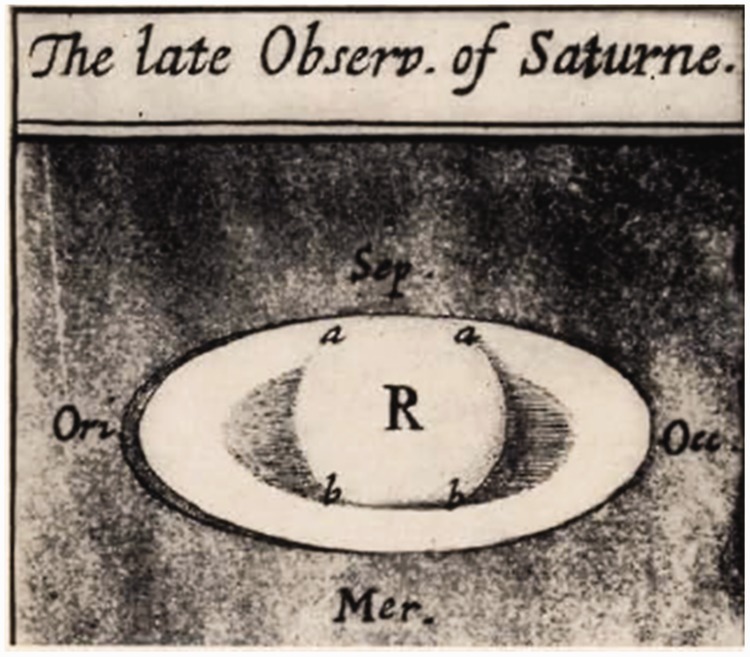
Drawing of Saturn by Robert Hook, taken from Philosophical Transactions (1666). Credit: Wikipedia Commons.

The old drawing of Hooke (1666) reproduced in Figure 8 makes evident the occlusion cues that allow to understand the relative position of the ring and the globe in space. The two cast shadows are illustrative: The right-hand letter *a* indicates the shadow of the globe on the ring, and the two letters *b* indicate the expected overlap of the rings on the globe.

*June 26 1666 between 11 and 12 at night I observed the body of Saturn through a 60 foot telescope and found it exactly of the shape represented in the figure. The ring appeared of a somewhat brighter light than the body, and the black lines crossing the ring and crossing the body (whether shadows or not, I dispute not) were plainly visible whence I could manifestly see, that the southernmost part of the ring was on this side of the body, and the northern part behind, or covered by the body. (Hooke, 1666, p. 245) (Credit: Leigh Fletcher,* *http://planetaryweather.blogspot.com/2013/03/early-views-of-saturn-galileo-and.html)*

That Hooke makes recourse to the analysis of the single depth cues and concludes with an inference that the figure represents a globe and an encircling ring, demonstrates that this visual pattern was not clear enough when the telescope was focused on the planet. But a further reason may be involved, something similar to mistrust about the perceived organization. The globe and the encircling ring are well distinguishable shapes and a thorough examination of the junctions should be superfluous unless suspicion arises that the image may be deceiving.

Several centuries later, a spacecraft bearing the name of one of the astronomers who enhanced our knowledge of Saturn (Cassini) sent an image of the planet that induced people not to believe what they were seeing. Besides being a picture of great astrophysical interest, it also has a very important significance for psychology, as it shows how Saturn and its rings are a fragile perceptual structure.

## Conclusion

The planet Saturn did not show its real “face” as an object which, emerging from the darkness or the haze, became increasingly defined in its detail and general appearance. The discovery of the planet is not the result of a progressive enrichment of knowledge. We have seen that the discovery took place, on Huygens’ part, on the basis of partial and discontinuous information. Instead, when sufficient information are collected, there will be resistance to accepting the idea that there is a celestial body surrounded by a large, flat ring. The planet has a peculiar “ability to hide itself.” My hypothesis is that this is in part due to the perceptual organization of the image of a globe surrounded by rings. It is a complex organization to discover where local clues (T-junctions) are required but also structural information. Huygens found the solution, albeit with partial information, because the graphic representation most likely allowed him to see the perceptual structure deriving from the formation of the double amodal completion. The disbelief of his contemporaries was due to the novelty of the “solution,” with resistance originating from rivals also playing an important part, but one wonders if, when looking at the drawings of Saturn and its rings, they struggled to “believe their own eyes.” In the outline sketch of the planet, reporting the whole set of clearly depicted depth cues (T-junctions), one has no difficulty to recognize the pattern; the problems arises when these pictorial cues blur or confound with the surrounding details as may happen in astronomic observation. It is enough for some details of the image of Saturn to be modified because, as the photo of the eclipse shows, the double amodal completion disappears and the image returns to the one that Galileo drew centuries ago. The eclipse of Saturn (Figure 1(b)) should not be considered a photographic trick but rather a stimulus to strengthen the emphasis of the perceptual organizations that we take for granted.

The disk planet is a homogeneous surface that can be perceived as an occluding object. This is not so for the ring, or component rings, because some of them are transparent, blurred, indistinguishable from their cast shadows, altered in luminance, and thickness at the interception with the globe contour. Also a close inspection of Saturn as the spacecraft Cassini made can give is a misleading image in which the amodal contour vanishes. The photo of Saturn Eclipse in Figure 1(b) illustrates the misleading effects of the rings contour alteration due to the particular photometric conditions in which the image was captured. A curious jump back in time caused by the most advanced astronomical observations.

A final remark to deal with an issue raised by an anonymous reviewer. Saturn, differently from other planets, elicits a three-dimensional impression, a figural organization in depth presumably originated from the rings occlusion. Tse (1998) created several demonstrations of illusory volumes, one of which (his Figure 10a) is—like Saturn—made up of concentric rings inclined in depth and interrupted, for some extent, by an invisible circular contour. The inner region has a “spherical appearance” according to the observers. The Saturn’s image exhibits phenomenological features that deserve great interest, in particular, the detailed photos of the planet released by Cassini in which the volumetric appearance of the globe emerges in a context of shadows and dark spaces.

## Notes

1 Historians who provides an analysis of the Galileo’s investigation: Favaro (1890–1909), Favaro (1900), and van Helden (1974b, p. 110).

2 From Systema Saturnium (translation by J. H. Walden, 1928), see http://www-history.mcs.st-andrews.ac.uk/Extras/Huygens_Saturn.html
*Furthermore, while I was considering these facts in connection with the motion of the arms, these arms appeared under the aspect which was exhibited at the time of my previous observations of the year 1655. The body of Saturn at its middle was quite round, while the arms extended on either side along the same straight line, as though the planet were pierced through the middle by a kind of axis; although, as indicated in the first figure of all, these arms, as seen through the twelve-foot telescope that I was then using, appeared a little thicker and brighter toward the ends on either side of the planet than they did where they joined the middle of the sphere. When, therefore, the planet continued day after day to present this same aspect, I came to understand that, inasmuch as the circuit of Saturn and the adhering bodies was so short, this could happen under no other condition than that the globe of Saturn were assumed to be surrounded equally on all sides by another body, and that thus a kind of ring encircled it about the middle; for so, with whatever velocity it revolved, it would always present the same aspect to us, if, of course, its axis were perpendicular to the plane of the ring.*

*And so was established the reason for the phase which continued through that period. Therefore, after that, I began to consider whether the other phases that Saturn was said to have could be accounted for by the same ring. I was not long in coming to a conclusion on this point through noting in frequent observations the obliquity of Saturn’s arms to the ecliptic. For when I had discovered that the straight line along which on. either side these arms projected did not follow the line of the ecliptic, but cut it at an angle of more than 20 degrees, I concluded that in the same way the plane of the ring which I had imagined was inclined at about the same angle to the plane of the ecliptic-with a permanent and unchanging inclination, be it understood, as is known to be the case on this Earth of ours with the plane of the equator. From this inclination it necessarily followed that in its different aspects the same ring showed to us at one time a rather broad ellipse, at another time a narrower ellipse, and sometimes even a straight line. As regards the handle-like formations, I understand that this phenomenon was due to the fact that the ring was not attached to the globe of Saturn, but was separated from it the same distance all around. These facts, accordingly, being thus brought into line, and the above-mentioned inclination of the ring being also assumed, all the wonderful appearance of Saturn, I found, could be referred to this source, as will presently be shown. And this is that very hypothesis which, in the year 1656, on the 25th day of March [earlier Huygens says the fifth of March, so one is a misprint, note by Walden], I put forth in confused letters, together with my observation on the Saturnian Moon.*

3 Fabri versus Huygens (credit: https://brunelleschi.imss.fi.it/esplora/cannocchiale/dswmedia/simula/esimula1_4_st.html).
